# Correction to ‘*Carbonodraco lundi* gen et sp. nov., the oldest parareptile, from Linton, Ohio, and new insights into the early radiation of reptiles’

**DOI:** 10.1098/rsos.192198

**Published:** 2020-01-22

**Authors:** Arjan Mann, Emily J. McDaniel, Emily R. McColville, Hillary C. Maddin

*R. Soc. open sci.* **6**, 191191 (Published Online 27 November 2019). (doi:10.1098/rsos.191191)

Our originally published manuscript ‘*Carbonodraco lundi* gen et sp. nov., the oldest parareptile, from Linton, Ohio, and new insights into the early radiation of reptiles’ did not include the required ZooBank accession number. This is supplied here:

LSIDurn:lsid:zoobank.org:pub:02676B44-D849-4ACC-8869-76D5460E4239

